# Financial burden of catastrophic health expenditure on households with chronic diseases: financial ratio analysis

**DOI:** 10.1186/s12913-022-07922-6

**Published:** 2022-04-27

**Authors:** Hyunwoo Jung, Young Dae Kwon, Jin-Won Noh

**Affiliations:** 1 Post Doc, Department of Health Administration, Graduate School·BK21 Graduate Program of Developing Glocal Experts in Health Policy and Management, Yonsei University, Wonju, South Korea; 2 Department of Humanities and Social Medicine, College of Medicine and Catholic Institute, for Healthcare Management, The Catholic University of Korea, Seoul, South Korea; 3 Division of Health Administration, College of Software and Digital Healthcare Convergence, Yonsei University, Wonju, South Korea

**Keywords:** Catastrophic health expenditure, Chronic disease, Financial strain

## Abstract

**Background:**

The financial status of households is vulnerable to chronic diseases which entail high medical expenses and income loss. Financial strain can be assessed by four indicators: a household surplus indicator, the liquid asset/debt ratio, a solvency indicator, and a liquidity indicator. We investigated the association between catastrophic health expenditure (CHE) and financial ratio indicators in households with chronic diseases in South Korea.

**Methods:**

This study applied thresholds to the financial ratios to determine the financial strain. We conducted multiple logistic regression analyses to determine whether CHE is associated with financial strain. Furthermore, we analyzed the relationship between CHE and basic financial indicators, absolute finance size, using multiple linear regression.

**Results:**

When CHE occurred, all financial ratio indicators deteriorated. However, this was not due to decreases in the absolute size of wealth and income, but rather the relative balance between finances. In particular, the loss of liquid assets was a major factor in the deterioration. In addition, all types of labor-related income deteriorated; only private transfer income increased.

**Conclusions:**

This study revealed that CHE in households with chronic diseases negatively impacts household finances. It was found that financial coping strategies are only resource consuming.

**Supplementary Information:**

The online version contains supplementary material available at 10.1186/s12913-022-07922-6.

## Background

South Korea (hereafter, Korea) is now classified as a high-income country and has achieved universal health coverage by implementing a national health insurance system. However, the incidence rate of catastrophic health expenditure (CHE) is high in Korea [1]. CHE is defined as an out-of-pocket (OOP) payment that is larger than a certain threshold of the household’s ability to pay [2]. The OOP means the share of health spending which patients should pay by themselves for health care services [1]. Many researchers have argued that Korean health insurance does not play a role in relieving OOP payments sufficiently because its benefit level is too low [3].

Chronic diseases have dominated the epidemiological landscape in Korea and around the world for decades [4]. Chronic diseases can affect household finances. First, direct costs, which are referred to as the expenses incurred when using health care services including OOP and other costs (transportation, special dietary regimes, etc.), can increase [5, 6]. Second, indirect costs can also increase. Indirect costs mean productive time losses, which can incur by reducing “working time” and “labor income” of the ill person and other household members [5, 6]. The economic impacts of chronic diseases include not only direct costs but also indirect costs.

To address these direct and indirect costs, households implement “financial coping strategies.” These strategies include the use of savings, borrowing from relatives or acquaintances, liability/loan borrowing, and the disposal of assets, which is defined as probable future economic benefits obtained or controlled by a particular entity as a result of past transactions or events, such as housing, land, and vehicles. Previous studies have argued that such financial strategies serve as buffers against economic crises [7–9].

However, simply taking out liability/loans and disposing of assets cannot prevent economic ruin and poverty. First, if one uses liability or borrowing without considering one’s ability to repay after a deterioration in health, it will lead to inextinguishable liability in the future [10]. If households fail to fulfill their obligations, they would become delinquent borrowers. Those labeled as such could not freely engage in social and economic activities and face various actions, including crime, that encourages liability fulfillment [11]. Second, Koreans usually regard assets as a means of bearing the costs of children’s education, marriage funding, maintaining life after retirement, and investment in asset growth. Therefore, if one disposed of assets due to spending on medical expenses, he would fail to achieve the objectives mentioned above and experience an economic burden. Furthermore, the disposal of assets makes the use of liability/loans more challenging because their availability varies depending on the size of the asset.

Many prior studies analyzing the impact of CHE on household finances were limited primarily to examining the effect of income on poverty. In the case of low- and middle-income countries, income could be a proxy for the whole household economy. However, in high-income countries, the sizes of assets and liability markets are also large, and only examining the effect on income is insufficient. We can use the basic financial indicators, which are comprised of each absolute size of assets, incomes, expenses, and liabilities in this situation [12, 13]. These indicators can evaluate the association between CHE and household finance more explicitly from multiple aspects.

However, finances in households are more complicated. Even if assets are substantial, an individual might go bankrupt when there is not enough cash to make the immediate repayments. To understand these complex effects, scholars in the personal finance field often suggest using financial ratio analysis [12–15]. Financial ratio analysis is a method that identifies the strengths and weaknesses of households through relative assessment and evaluates whether households are doing well in achieving their financial goals over time by using several financial ratio indicators [16, 17]. The Methods section will explain the specific indicators.

Therefore, this study examines whether CHE is associated with the increase of financial strain in households with chronic disease, and financial coping strategies would prove only to consume more resources. Furthermore, to understand better how chronic disease impacts indirect costs, we classified income types as labor-related and non-labor-related and analyzed them. To achieve this objective, we conducted several analyses, as follows.

- Compared the incidence rate of CHE in households with and without chronic diseases.
  - ▪ Hypothesis 1: The incidence rate of CHE is higher in households with chronic diseases than in those without.

- Analyzed the association between CHE and several financial ratio indicators: a surplus indicator, the liquid asset/debt ratio (LADR), a solvency indicator, and a liquidity indicator, which are measured by the ratio of two basic financial indicators (among the absolute size of financial assets, liability, income, and living expenses). See the Methods section for descriptions of the specific indicators and calculation methods.
  - ▪ Hypothesis 2: There are associations between CHE and the deterioration in financial ratio indicators.

- Analyzed the association between CHE and several basic financial indicators and each type of income (Labor-related or non-related).
  - ▪ Hypothesis 3: There are associations between CHE and the deterioration in basic financial indicators.
  - ▪ Hypothesis 4: There are associations between CHE and the decrease in labor-related incomes.
  - ▪ Hypothesis 5: There is no association between CHE and non-labor-related incomes.

## Methods

### Data

This study utilized data from the Korea Welfare Panel Study (KoWePS), which covers all cities and provinces in Korea. The KoWePS evaluates samples using a two-stage stratified cluster sampling design and collects data from household financial records and receipts of OOP to avoid recall bias. The KoWePS provides various data, such as demographic and socioeconomic characteristics of individuals, OOP, assets, liabilities, and income.

We mainly used data from 2014, but we added data from 2015 and 2016 as needed. Specifically, since there may have been reverse causality between the independent and dependent variables, we first set the independent variable as the data from 2014 and the dependent variable as the data from 2015. Second, the KoWePS collects data from the relevant year; however, when it comes to the income and living expense variables, it investigates data from the previous year. This is because the KoWePS survey period is so long that the response time of each subject varies. Some may have been investigated in May and others in September. In addition, if the survey is conducted in May, income and living expenses cannot represent for one full year. However, other variables, such as assets or liability, gender, and education level, do not need to consider response time, so it investigated the data of the relevant year. Therefore, we conducted time matching. For example, as mentioned above, we set the independent variable (CHE) as the data from 2014 and the dependent variable (i.e., a surplus indicator) as the data from 2015. When calculating CHE, we used the OOP variable of 2014 and the income variable from 2015. At the same time, when calculating the surplus indicator, we used the living expenses and income variable from 2016.

The subjects of this study were households with chronic diseases. The KoWePS collects data on whether they have such diseases or take medicine for more than six months. If at least one person had one or more chronic diseases, we defined the household to which that person belonged as a chronic disease household. Accordingly, 6,270 households participated in the survey for three years, and 4,802 households had chronic diseases.

We used publicly available and reliable secondary data from the KoWePS, which were provided through the de-identified samples. The investigator of the KoWePS visited the participants’ households, met the respondents, and gained informed consent. Approval of IRB exemption for this study was granted by the Korea University Institutional Review Board (approval number: KUIRB-2019–0214-01), given the retrospective nature of the study.

## Measures of CHE

We calculated the independent variable CHE according to the method of Wagstaff and van Doorslaer (2003) [14]. We divided OOP by household payment ability (disposable income–food expenses) and transformed it into a binary variable [18, 19]. When its value was greater than the threshold (i.e., 10%, 20%, 30%, or 40%), CHE was Yes (1), and No (0) otherwise. In the multiple logistic and linear analyses, we used a representative threshold of 10% because it is the approximate level at which households would sell their assets to pay for OOP, pay off liability, and reduce the cost of living other than health expenditure [20]. The threshold level is a commonly used value in empirical studies conducted to date [1].

The OOP of the KoWePS included costs for hospitalization, outpatient care, dental care, surgery (including implants and cosmetic surgery), medicine, nursing care, postpartum care, health checkups, and healthcare supplies (eyeglasses, contact lenses, etc.).

## Measures of financial strain indicators

There are two types of financial strain indicator for households: basic financial indicators and financial ratio indicators [12, 13]. First, basic financial indicators are comprised of the absolute size of each financial component: disposable income, earned income, total living expenses with and without OOP, savings, total assets, liquid assets, non-liquid assets, and the total liabilities of households [13, 21]. In terms of income, we classified it as labor-related income (earned and business incomes) and non-labor income (property, private transfer, public transfer, and other incomes) to determine whether CHE in households with chronic disease conferred a detrimental effect on individuals’ work activities.

The basic financial indicators facilitate objective comparison and evaluation; however, they limit assessing the complex aspects of household financial conditions. A financial ratio indicator allows us to better understand these complexities [14]. Financial ratio indicators are measured as the ratio of two financial elements, and a threshold can be set to determine whether there is a financial strain. As financial advisors recommend using several ratios [14], we included the following four indicators:

### Surplus indicator

The surplus indicator is a primary indicator for evaluating the adequacy of household cash flow that can help determine whether the consumption propensity is fine and whether there is the possibility of a deficit in a household [22]. In this study, we measured the surplus indicator as the annual “total living expenses/disposable income” and applied a threshold of 70%, based on Yang et al. (2013) [13]. If the value exceeded 70%, we categorized it as “dangerous.”

#### LADR

The LADR is the concept of the ability to liquidate households’ total debts with their liquid assets. Liquid assets include deposit and installment savings of bank and stock bonds. Total assets include liquid assets and non-current assets, such as real estate (houses, non-residential buildings, land), agricultural machinery, livestock products (cows, pigs, chickens, etc.), and current car quotations. We defined the bad condition of LADR as liquid assets lower than 20% of their liabilities, as suggested by Griffith (1985) [23].

### Solvency indicator

The solvency indicator is an indicator of a household's overall financial condition, which determines whether they have total assets to sustain their debts [17, 22]. This indicator is represented by “total assets/total liabilities.” Total liabilities include loans from financial institutions, general debentures, card debt, credit, and deposits on leases. We defined this as dangerous if the ratio was over 40% [13, 17, 22].

### Liquidity indicator

The liquidity indicator assesses whether liquid assets can sustain the previous standard of living when income is temporarily suspended [13]. It can show whether cash flows and assets are well balanced. It is calculated as “liquid assets/disposable income” and is classified as a bad condition if the ratio is lower than 400%, based on DeVaney (1993) and Yang et al. (2013) [12, 17].

## Control variables

We chose the control variables based on previous studies [24]: household characteristics (number of household members, type of medical insurance, presence of disabled individuals, children younger than 20, elderly individuals aged 65 or older), and the characteristics of the householders (gender, age group, educational level, marital status, employment status). Since the independent and dependent variables in this study were household units, we did not include health-related variables, such as the type of disease and self-reported health, because these are individual units.

## Statistical analysis

This study performed three analyses. First, we compared the CHE incidence rates in households with and without chronic diseases using the chi-square test. At this time, we represented various thresholds (10%, 20%, 30%, and 40%) to examine the sensitivity of CHE incidence. Second, we conducted multiple logistic regression analyses to identify the relationship between CHE and financial ratio indicators in households with chronic diseases. We transformed every dependent variable (financial ratio) to a binary variable, as good (0) or bad (1), according to the thresholds suggested in previous studies.

However, since the financial ratio was calculated as a ratio, it was necessary to provide further shape to the model [25]. It is difficult to determine whether the increase in the proportion of the surplus indicator is due to decreased income or increased living costs [14]. Third, we used multiple linear regression analyses to analyze the relationship between CHE and basic financial indicators. All the basic financial indicators often had a non-Gaussian distribution, and because the distribution of the error term is unlikely to be a normal distribution, we applied a logarithmic transformation [14, 25].

The variance inflation factor (VIF) was used to test for multicollinearity among the independent variables. Because all the values of the VIF were less than 10, multicollinearity could be ignored. To resolve heteroskedasticity, we computed robust standard errors (detailed explanations are available at https://www.stata.com/search/). We used the statistical software program Stata version 14.0 (StataCorp, College Station, Texas, US).

## Results

### General characteristics

The characteristics of the samples are presented in Table 1. It is notable that most households were unemployed (46.1%), and most of the households had elderly residents (67.4%). These results would have stemmed from the characteristics related to chronic diseases in the KoWePS; as in the total sample of 6,270, the unemployment rate was already 36.1%, and 55.1% of households had an elderly resident. Additionally, in the results for the type of national health insurance, employees totaled 63%, which contrasts with the unemployed rate of the household head. This can be interpreted as many household heads being retired and other household members working instead (Table 1).

**Table 1:** Sample characteristics

Variables			Number of households with chronic diseases	%	Total number of households
Characteristics of household head	Gender	Male	3,208	66.8	4,381
		Female	1,594	33.2	1,889
	Age group	20–39	1,283	26.7	1,765
		40–64	1,755	36.6	2,656
		65 or older	1,764	36.7	1,849
	Educational level	Less than elementary school	802	16.7	1,469
		Middle-high school	1,984	41.3	2,615
		Greater than college	2,016	42.0	2,186
	Marital status	Married	2,794	58.2	3,800
		Single	2,008	41.8	2,470
	Employment status	Employee	1,329	27.7	2,287
		Employer/owner	1,124	23.4	1,413
		Other	133	2.8	149
		Unemployed	2,216	46.1	2,421
Characteristics of household	Number of household members	1	1,438	29.9	1,758
		2	1,795	37.4	2,051
		3	683	14.2	1,029
		4 or more	886	18.5	1,432
	Type of national health insurance	Employee	3,028	63.0	4,026
		Self-employed	1,268	26.4	1,687
		Medical aid beneficiaries	506	10.6	557
	Private health insurance	Insured	2,327	48.5	3,549
		Uninsured	2,475	51.5	2,721
	Presence of disabled person	No	4,377	91.2	5,819
		Yes	425	8.8	451
	Presence of children	No	3,935	82.0	4,690
		Yes	867	18.0	1,580
	Presence of elderly	No	1,564	32.6	2,812
		Yes	3,238	67.4	3,458

Presence of children indicates those under 20 years old; presence of elderly means those 65 years or older; widows/widowers are included as single in marital status due to the small number of cases; in employment status, other includes senior/disabled/retired people who participated in the government project for employment promotion, as well as unpaid family workers

## Incidence rates of CHE

The incidence rates of CHE are shown in Table 2. Rates were higher in households with chronic disease. For a typical threshold of 10%, the CHE incidence was only 6.5% in households without chronic disease, while it was 4.4 times higher in households with chronic disease (28.9%). The CHE incidence rates in households with chronic diseases were also high at all thresholds. In addition, thresholds greater than 20% were not suitable for various analyses because of the small sample size (Table 2).

**Table 2:** Different incidence rates of CHE between households with and without chronic disease

Variables		Households without chronic disease		Households with chronic disease		Total	*P*-value
		**Frequency**	**%**	**Frequency**	**%**		
**CHE (10%)**	No	1,372	93.5	3,414	71.1	4,786	< 0.001
	Yes	96	6.5	1,388	28.9	1,484	
**CHE (20%)**	No	1,437	97.9	4,125	85.9	5,562	< 0.001
	Yes	31	2.1	677	14.1	708	
**CHE (30%)**	No	1,459	99.4	4,451	92.7	5,910	< 0.001
	Yes	9	0.6	351	7.3	360	
**CHE (40%)**	No	1,464	99.7	4,602	95.8	6,066	< 0.001
	Yes	4	0.3	200	4.1	204	

*CHE* catastrophic health expenditure

## Association between CHE and financial strain in households with chronic disease

Table 3 shows the association between CHE and financial strain, analyzed using multiple logistic and linear regression analyses. When CHE (threshold 10%) occurred, all financial ratio indicators (surplus, liquidity, solvency indicator, and LADR) deteriorated significantly. In addition, there were differences among the basic financial indicators.

**Table 3:** Effect of CHE on financial strain indicators in households with chronic disease

Independent variable	CHE (OOP/Disposable income > 0.1)					
Dependent variables	**OR (SE)**	**Coefficient (RSE)**	**Constant (RSE)**	**N**	**Pseudo R**^**2**^ **(Adj R**^**2**^**)**	**LR chi**^**2**^
Surplus indicator (TLE / Income > 0.7)	3.248^**^ (0.557)		3.468^**^ (0.971)	4,802	0.118	344.8
Total living expense		0.058^**^ (0.014)	7.869^**^ (0.035)	4,802	(0.717)	
Disposable income		-0.164^**^ (0.017)	7.951^**^(0.04)	4,783	(0.658)	
LADR (Liquid asset / total liability < 0.2)	1.301^*^ (0.133)		0.069^**^ (0.015)	4,802	0.055	222.4
Liquid asset		-0.383^**^ (0.069)	8.438^**^ (0.164)	4,802	(0.274)	
Total liability		0.007 (0.123)	3.767^**^ (0.294)	4,802	(0.182)	
Solvency indicator (Total asset / total liability > 0.4)	1.448^*^ (0.167)		0.089^**^ (0.021)	4,802	0.055	222.4
Total asset		-0.146^*^ (0.056)	10.04^**^ (0.135)	4,802	(0.373)	
Total liability		0.007 (0.123)	3.767^**^ (0.294)	4,802	(0.182)	
Liquidity indicator (Liquid asset / income < 4)	1.375^*^ (0.217)		0.012^**^ (0.004)	4,802	0.067	129.04
Liquid asset		-0.383^**^ (0.069)	8.438^**^ (0.164)	4,802	(0.274)	
Disposable income		-0.164^**^ (0.017)	7.951^**^(0.04)	4,783	(0.658)	
Supplementary						
Non-liquid asset		-0.036 (0.049)	9.736^**^ (0.115)	4,295	(0.388)	
Total living expense without OOP		-0.096^**^ (0.014)	12.78^**^ (0.034)	4,802	(0.743)	

*CHE* Catastrophic health expenditure, *OR* odds ratio, SE standard error, *OOP* out-of-pocket, *RSE* robust standard error, *TLE* total living expense, *LADR* liquid asset/debt ratio

Results from four logistic and nine linear regressions separately; all regressions’ prob > chi^2^, F are < 0.001; all the models included covariates as presented in the additional files

^**^p < 0.001

^*^p < 0.05

First, the CHE had an odds ratio (OR) of 3.248 (p < 0.000) on the surplus indicator, which means that the indicator deteriorated. Further, as a result of further analysis to identify the cause of the deterioration in the indicators, CHE significantly decreased the logarithm of disposable income (coefficient = -0.164; this means that 15.1% of income decreased when converted from logarithm) and increased the total living costs (coefficient = 0.058; this means a 6.0% increase). In this case, the ratio value increased because of the decreasing denominator and increasing numerator. Moreover, we analyzed the logarithm of the cost of living, excluding OOP, as a dependent variable. As a result, it decreased significantly (coefficient = -0.096; this means a 9.2% reduction) when CHE occurred (below the supplementary cell).

Second, CHE correlated with the deterioration of the LADR (OR = 1.301; *p* < 0.05), which suggests that CHE weakens a household’s capacity to hold liability. CHE did not affect total liability, but significantly reduced liquid assets (coefficient = -0.383; 31.8% decrease).

Third, CHE was related with a worsening of the solvency index (OR = 1.448; *p* < 0.01), which may have aggravated household finances in the long term and at the macro level. This indicator is composed of total liabilities and assets. CHE did not affect total liabilities but lowered the logarithm of total assets (coefficient = -0.146; this means a 13.6% decrease). We further classified the types of assets into liquid and non-liquid assets to identify how asset reduction occurs. As a result, CHE had no significant impact on non-liquid assets. Instead, CHE was associated with reduced liquid assets.

Fourth, CHE correlated with the exacerbation of the liquidity indicator as much as an OR of 1.375. This indicator is the ratio of disposable income to liquid assets. Since CHE conferred a negative effect on both sides, it was difficult to determine which one had an impact. However, it seems that the decrease in liquid assets is more considerable than income (disposable income coefficient = -0.164; 15% decrease, liquid asset coefficient = -0.383; 31.8% reduction) (Table 3).

Table 4 shows that even in households with chronic diseases, CHE still correlated with the decrease in labor-related income, which is “earned income” (coefficient = -0.385; this means a 32% decrease) and “business income” (coefficient = -0.418; this means a 34.2% decrease). On the other hand, of all income types, the only increase occurred in private transfer income (coefficient = 0.41; this means a 50.7% increase). On the contrary, public transfer income, which is paid by the social security system, decreased inversely (coefficient = -0.143; this means a 13.4% decrease) (Table 4).

**Table 4:** Effect of CHE on each type of income

	Dependent variables (Log)											
	Labor-related income				Non-labor-related income							
	Earned income		Business income		Property income		Private transfer income		Public transfer income		Other income	
	Coef	RSE	Coef	RSE	Coef	RSE	Coef	RSE	Coef	RSE	Coef	RSE
CHE	-0.385^**^	0.044	-0.418^**^	0.075	-0.106	0.087	0.410^**^	0.048	-0.143^**^	0.030	0.309	0.064
Constant	7.450**	0.081	6.057^**^	0.216	5.172^**^	0.244	4.791^**^	0.116	5.749^**^	0.078	2.685^**^	0.152
N		2,467		1,627		1,797		4,781		4,112		4,709
Prob > F		0.000		0.000		0.000		0.000		0.000		0.000
Adj R-squared		0.607		0.553		0.149		0.217		0.179		0.131

*CHE* catastrophic health expenditure

Results from six different regressions; included covariates are the same as in Table 3

^**^p < 0.001

^*^p < 0.05

## Discussion

The purpose of this study was to determine whether households with chronic diseases are vulnerable to CHE and to examine whether CHE is associated with the increase of financial strain in households with chronic diseases. Consequently, the incidence rate of CHE was found to be higher in households with chronic disease than in those without as presented in Table 2. This is because households with chronic diseases incur a large OOP, and their income decreases because of health deterioration. In Korea, specifically, since the national health insurance coverage is not sufficient, CHE is highly likely to occur in households with chronic diseases [3].

Furthermore, when CHE occurred in households with chronic disease, almost all financial strain indicators deteriorated in the following year. This study involved two main analyses. First, we conducted logistic regressions to evaluate the financial ratio indicators applied to the threshold model. Second, we analyzed linear regression to determine the components of the ratio indicators affected.

We analyzed four financial ratio indicators: surplus, LADR, solvency, and liquidity. To summarize the results, the surplus indicator deteriorated. This could interrupt cash flow. In an additional analysis, it was found that income loss and an increase in living costs affected the surplus indicator. If households with chronic diseases have CHE, the patient may not be able to work, and even other household members may give up work to take care of the patient, which could reduce their income. Furthermore, it seems that medical expenses increased the total living costs. However, the total living expenses without OOP decreased. It can be interpreted that households reduce other consumption to pay for OOP.

Second, the LADR deteriorated, which means that CHE weakens a household’s capacity to hold liability. The LADR is an assessment of the amount of liability that can be repaid through liquid assets that can be mobilized immediately in the event of an unavoidable situation in which a household must repay all of its liability at once [13, 23, 26]. LADR is the ratio of total liabilities to liquid assets. As a result of the further analysis, CHE did not affect total liability, but significantly reduced liquid assets. It can be interpreted that households pay for medical costs by withdrawing liquid assets and not borrowing money from the bank. The decline in liquid assets can hinder cash flow, making expenses more difficult and increasing the burden of paying off liabilities [19, 27].

The solvency indicator had an enormous financial scale compared to the other indicators, but the results showed that it associated with CHE. This indicator reflects the full capacity to hold household liabilities, so that it should be alert. Further analysis showed that CHE had no significant relationship with non-liquid assets. Instead, CHE was associated with reduced liquid assets. It can be interpreted that CHE occurs suddenly, and individuals rely on financial assets available for immediate use. Many people view the use of liquid assets as a financial coping strategy, but it is not, as this will eventually lead to a decline in household finances.

Finally, the liquidity indicator also deteriorated, which means that households lose the ability to cope with unexpected economic problems. The increase in the liquidity indicator indicates that the money needed to maintain the present economic condition will disappear. In this case, households would become very unstable, both psychologically and economically. A further analysis to identify the cause showed that CHE conferred a negative effect on both sides; however, the decrease in liquid assets was higher.

From the results of the four financial ratio analyses, we can deduce several facts, as follows: First, the likelihood of household financial strain is high if CHE occurs in households with chronic diseases. This is not due to a decrease in absolute wealth or income or an increase in debt but rather because CHE broke the relative balance among financial elements. Second, the main factor in the deterioration of the financial ratio index was a decrease in liquid assets. This can be interpreted as people using liquid assets rather than debt or non-liquid assets when CHE occurs. Since three of the four indicators (LADR, solvency, and liquidity indicators) contain liquid assets, this affects all indicators in a chain. Liquid assets and income are different. Income is “newly incoming money,” and liquid assets are “accumulated money” through income. For example, if an employee uses an outpatient service or even pays for expensive medicine, it will not reduce the incoming money (i.e., the salary) but reduce the accumulated money. If OOP were to affect income, then it might be the case that a patient's illness (OOP implied) is so severe that they may not be able to maintain work activities and will lose their wages. Previous studies that analyzed only the impact of CHE on income-based poverty did not point out any differences between incoming and accumulated money.

Third, CHE correlated with the increase in the total cost of living and decreased income, which led to the deterioration of the surplus indicator. Previous studies used the surplus indicator as a tool for short-term evaluation [12, 13]; however, the deterioration of the indicator can develop into a long-term problem when it comes to chronic diseases because it implies a loss of workability. To determine whether income reduction occurs through labor, we analyzed the association between CHE and income type (labor-related and non-labor-related).

As a result, labor-related income declined. Since CHE represents a more severe status of chronic diseases, it can be interpreted that CHE negatively affects the workability of household members. This interpretation is supported by the fact that non-labor-related income was not affected. Of all income types, the only increase occurred in private transfer income. This can be considered as borrowing money from nearby acquaintances to offset OOP and reduce earned income. In other words, Korean people rely more heavily on social networks than the social security system, which must protect household finances by providing public transfer income. However, private transfer income may not be sufficient to cover the decreasing disposable income. In addition, even if a household reduces expenditure on other goods or services, the total living expenses remain elevated because of OOP. As living expenses increase and income decreases, household cash flows show a deficit.

## Limitations

This study has some limitations. This study could not present relative risks by analyzing modified Poisson regression. Relative risks have an advantage in interpreting more intuitively than the OR. However, it was difficult to use because the prerequisites for the causal relationship of Poisson regression could not be met. This study has a limitation in that it cannot analyze whether CHE has a causal effect on financial indicators, only the correlation investigated. To analyze the causal impact, it is necessary to measure the difference in financial indicators before and after the occurrence of CHE. However, it is difficult to calculate the difference because the financial indicators are measured as a ratio. Moreover, there is a problem that selection bias may occur as the number of samples decreases during the calculation process. In addition, the relationship between the CHE indicator and surplus indicator could have endogeneity. For example, households with a strong consumption propensity will be high in both indicators, and households with a low consumption propensity will be low.

Furthermore, this study used the family units of the independent and dependent variables. Therefore, we could not use individual units of the control variables, such as self-reported health. We suggest that subsequent studies utilize panel analysis, considering that chronic diseases affect people over the long term. In particular, liability may have a positive impact on maintaining consumption in households in the short term, but it would likely become an economic burden to repay over the long term. In other words, since the economic burden of liability occurs over time, we must observe a long-term impact. Moreover, income, assets, and liabilities are influenced by the characteristics of households that are not observed; however, a panel analysis could control for such effects.

## Conclusions

Industrialization and globalization have created large economies worldwide alongside aging of the world population as a whole. Accordingly, we are entering the era of chronic disease pandemics [4, 28–30]. Chronic diseases are challenging to cure, leading to an economic crisis for households over a long period. In addition, advances in medical technology and pharmaceutical innovation due to industrialization and globalization have accelerated medical expenses [31]. In the past, chronic diseases were regarded as “prosperity” diseases in advanced countries [31]. Now, however, they are permeating middle- and low-income countries, so that medical expenses are expected to skyrocket in the future worldwide [32]. Therefore, the issue of chronic disease is not limited to Korea but affects the whole global population, and it is important to deal wisely with financial strain brought on by diseases.

Existing studies argue that exploiting liability, borrowing, and using savings is a financial coping strategy that can protect households from poverty [8, 9]; however, this strategy breaks the balance of financial fitness in households. In this study, we demonstrated that several financial strain indicators have deteriorated. These are important facts for an economically advanced country like Korea because one of the reasons for the bankruptcy of households in the Korean community is that they fail to balance the liability/assets ratio. There was no significant increase in liability in this study; however, the relative size of assets could be more critical than the absolute liability size. Even a small liability, if a household is incapable of servicing it, will cause an over liability situation [10]. If assets are reduced, their ability to maintain liability decreases. Households can be classified as credit-impaired in the event of a default and may become bankrupt [33]. A financial ratio analysis was conducted to understand this point.

## Supplementary Information

**Additional file 1:** **Supplementary table 1.** Effect of catastrophic healthexpenditure on surplus indicator.

**Additional file 2: Supplementary table 2.** Effect of catastrophic health expenditure on solvency indicator.

**Additional file 3: Supplementary table 3.** Effect of catastrophic health expenditure on LADR.

**Additional file 4: Supplementary table 4.** Effect of catastrophic health expenditure on liquidity indicator.

**Additional file 5: Supplementary table 5.** Effect of catastrophic health expenditure on total living expenses.

**Additional file 6: Supplementary table 6.** Effect of catastrophic health expenditure on disposable income.

**Additional file 7: Supplementary table 7.** Effect of catastrophic health expenditure on liquid assets.

**Additional file 8: Supplementary table 8.** Effect of catastrophic health expenditure on total liability.

**Additional file 9: Supplementary table 9.** Effect of catastrophic health expenditure on total assets.

**Additional file 10: Supplementary table 10.** Effect of catastrophic health expenditure on non-liquid assets.

**Additional file 11: Supplementary table 11.** Effect of catastrophic health expenditure on total living expenses without OOP

**Additional file 12: Supplementary table 12.** Effect of catastrophic health expenditure on earned income.

**Additional file 13: Supplementary table 13.** Effect of catastrophic health expenditure on business income.

**Additional file 14: Supplementary table 14.** Effect of catastrophic health expenditure on property income.

**Additional file 15: Supplementary table 15.**Effect of catastrophic health expenditure on private transfer income.

**Additional file 16: Supplementary table 16.** Effect of catastrophic health expenditure on public transfer income.

**Additional file 17: Supplementary table 17.** Effect of catastrophic health expenditure on other income.

**Additional file 18.** irb report.

**Additional file 19.** Thedatabase link of Korea Welfare Panel Study (KoWePS).

**Additional file 20.** Financial burden of CHE analysis.

## Data Availability

We used publicly available and reliable secondary data from the KoWePS, which were provided through de-identified samples. The investigator of the KoWePS visited the participants’ households, met the respondents, and obtained informed consent.

## Abbreviations

CHE: Catastrophic Health Expenditure
OOP: Out-of-pocket
KoWePS: Korea Welfare Panel Study
LADR: Liquid Asset/Debt Ratio
VIF: Variance Inflation Factor
OR: Odds Ratio
