# Correction: Financial burden of catastrophic health expenditure on households with chronic diseases: financial ratio analysis

**DOI:** 10.1186/s12913-022-08829-y

**Published:** 2022-11-17

**Authors:** Hyunwoo Jung, Young Dae Kwon, Jin-Won Noh

**Affiliations:** 1 Post Doc, Department of Health Administration, Graduate School·BK21 Graduate Program of Developing Glocal Experts in Health Policy and Management, Yonsei University, Wonju, South Korea; 2 Department of Humanities and Social Medicine, College of Medicine and Catholic Institute, for Healthcare Management, The Catholic University of Korea, Seoul, South Korea; 3 Division of Health Administration, College of Software and Digital Healthcare Convergence, Yonsei University, Wonju, South Korea

**Correction to: BMC Health Serv Res 22, 568 (2022)**

**https://doi.org/10.1186/s12913-022-07922-6**

Following publication of the original article [1], the authors reported an error in the ‘Funding’ section.

The statement in the ‘Funding’ section originally read: This research was supported by a grant of Patient-Centered Clinical Research Coordinating Center funded by the Ministry of Health & Welfare, Republic of Korea (grant number: HI19C0481, **H21C0059**).

The statement in the ‘Funding’ section should read: This research was supported by a grant of Patient-Centered Clinical Research Coordinating Center funded by the Ministry of Health & Welfare, Republic of Korea (grant number: HI19C0481, **HC21C0059**).

The original article [1] has been updated.
