# Supplementary material for: Financial burden of catastrophic health expenditure on households with chronic diseases: financial ratio analysis
Source: BMC Health Serv Res. 2022 Apr 27;22:568. doi: 10.1186/s12913-022-07922-6 (PMC9047277; doi:10.1186/s12913-022-07922-6)
Supplement: Supplementary file 1 — Additional file 1: Supplementary table 1. Effect of catastrophic healthexpenditure on surplus indicator. [file 12913_2022_7922_MOESM1_ESM.docx]

Supplementary table 1. Effect of catastrophic health expenditure on surplus indicator

|  | | Odds Ratio | S.E. | P>\|z\| |
| --- | --- | --- | --- | --- |
| CHE | | 3.248 | 0.556 | 0.000 |
| Gender (Men) | | 1.547 | 0.326 | 0.039 |
| Age  (<39) | 40~64 | 1.313 | 0.230 | 0.119 |
|  | >65 | 1.335 | 0.229 | 0.092 |
| Educational level  (Elementary school) | Middle-high school | 1.191 | 0.167 | 0.213 |
|  | Greater than college | 1.281 | 0.229 | 0.166 |
| Marital (married) | Divorced, bereavement, separation | 0.810 | 0.222 | 0.445 |
|  | Unmarried | 0.846 | 0.185 | 0.447 |
| Employment  (Employee) | Employer/  Self-employed | 0.868 | 0.123 | 0.323 |
|  | Other | 0.733 | 0.247 | 0.358 |
|  | Unemployed | 1.588 | 0.257 | 0.004 |
| No. of household members (1) | 2 | 0.547 | 0.119 | 0.006 |
|  | 3 | 0.466 | 0.115 | 0.002 |
|  | >4 | 0.600 | 0.170 | 0.072 |
| Type of NHI  (Employee) | Employer/  Self-employed | 1.126 | 0.142 | 0.346 |
|  | Medical aid beneficiaries | 2.533 | 0.866 | 0.007 |
| Private insurance  (Insured) | Uninsured | 1.651 | 0.244 | 0.001 |
| Presence of disabled (No) | Yes | 1.394 | 0.393 | 0.239 |
| Presence of child (No) | Yes | 3.062 | 0.545 | 0.000 |
| Presence of elderly (No) | Yes | 1.694 | 0.298 | 0.003 |
| Constant | | 3.468 | 0.971 | 0.000 |
| N | | 4,802 | | |
| Log likelihood | | -1277.6 | | |
| Pseudo R2 | | 0.119 | | |
