# Supplementary material for: Financial burden of catastrophic health expenditure on households with chronic diseases: financial ratio analysis
Source: BMC Health Serv Res. 2022 Apr 27;22:568. doi: 10.1186/s12913-022-07922-6 (PMC9047277; doi:10.1186/s12913-022-07922-6)
Supplement: Supplementary file 2 — Additional file 2: Supplementary table 2. Effect of catastrophic health expenditure on solvency indicator. [file 12913_2022_7922_MOESM2_ESM.docx]

Supplementary table 2. Effect of catastrophic health expenditure on solvency indicator

|  | | Odds Ratio | S.E. | P>\|z\| |
| --- | --- | --- | --- | --- |
| CHE | | 1.448 | 0.167 | 0.001 |
| Gender (Men) | | 0.889 | 0.138 | 0.454 |
| Age  (<39) | 40~64 | 0.859 | 0.130 | 0.321 |
|  | >65 | 0.640 | 0.094 | 0.003 |
| Educational level  (Elementary school) | Middle-high school | 1.324 | 0.172 | 0.031 |
|  | Greater than college | 0.799 | 0.133 | 0.181 |
| Marital (married) | Divorced, bereavement, separation | 1.298 | 0.324 | 0.296 |
|  | Unmarried | 1.948 | 0.338 | 0.000 |
| Employment  (Employee) | Employer/  Self-employed | 0.456 | 0.067 | 0.000 |
|  | Other | 0.462 | 0.167 | 0.034 |
|  | Unemployed | 0.713 | 0.099 | 0.016 |
| No. of household members (1) | 2 | 1.312 | 0.213 | 0.094 |
|  | 3 | 1.857 | 0.383 | 0.003 |
|  | >4 | 1.998 | 0.484 | 0.004 |
| Type of NHI  (Employee) | Employer/  Self-employed | 1.723 | 0.195 | 0.000 |
|  | Medical aid beneficiaries | 3.450 | 0.570 | 0.000 |
| Private insurance  (Insured) | Uninsured | 1.018 | 0.132 | 0.885 |
| Presence of disabled (No) | Yes | 1.121 | 0.181 | 0.477 |
| Presence of child (No) | Yes | 1.575 | 0.230 | 0.002 |
| Presence of elderly (No) | Yes | 0.633 | 0.095 | 0.002 |
| Constant | | 0.089 | 0.021 | 0.000 |
| N | | 4,802 | | |
| Log likelihood | | -1595.9 | | |
| Pseudo R2 | | 0.097 | | |
