# Supplementary material for: Financial burden of catastrophic health expenditure on households with chronic diseases: financial ratio analysis
Source: BMC Health Serv Res. 2022 Apr 27;22:568. doi: 10.1186/s12913-022-07922-6 (PMC9047277; doi:10.1186/s12913-022-07922-6)
Supplement: Supplementary file 3 — Additional file 3: Supplementary table 3. Effect of catastrophic health expenditure on LADR. [file 12913_2022_7922_MOESM3_ESM.docx]

Supplementary table 3. Effect of catastrophic health expenditure on LADR

|  | | Odds Ratio | S.E. | P>\|z\| |
| --- | --- | --- | --- | --- |
| CHE | | 1.301 | 0.132 | 0.01 |
| Gender (Men) | | 0.848 | 0.124 | 0.261 |
| Age  (<39) | 40~64 | 1.081 | 0.154 | 0.580 |
|  | >65 | 0.668 | 0.083 | 0.001 |
| Educational level  (Elementary school) | Middle-high school | 1.370 | 0.165 | 0.009 |
|  | Greater than college | 0.916 | 0.136 | 0.558 |
| Marital (married) | Divorced, bereavement, separation | 1.058 | 0.250 | 0.810 |
|  | Unmarried | 1.503 | 0.241 | 0.011 |
| Employment  (Employee) | Employer/  Self-employed | 0.730 | 0.093 | 0.014 |
|  | Other | 0.587 | 0.191 | 0.103 |
|  | Unemployed | 0.941 | 0.118 | 0.635 |
| No. of household members (1) | 2 | 1.554 | 0.236 | 0.004 |
|  | 3 | 2.068 | 0.395 | 0.000 |
|  | >4 | 2.634 | 0.589 | 0.000 |
| Type of NHI  (Employee) | Employer/  Self-employed | 1.624 | 0.162 | 0.000 |
|  | Medical aid beneficiaries | 2.553 | 0.394 | 0.000 |
| Private insurance  (Insured) | Uninsured | 1.097 | 0.125 | 0.419 |
| Presence of disabled (No) | Yes | 0.988 | 0.149 | 0.941 |
| Presence of child (No) | Yes | 1.206 | 0.162 | 0.163 |
| Presence of elderly (No) | Yes | 0.982 | 0.134 | 0.900 |
| Constant | | 0.069 | 0.015 | 0.000 |
| N | | 4,802 | | |
| Log likelihood | | -1902.4 | | |
| Pseudo R2 | | 0.055 | | |
