# Supplementary material for: Financial burden of catastrophic health expenditure on households with chronic diseases: financial ratio analysis
Source: BMC Health Serv Res. 2022 Apr 27;22:568. doi: 10.1186/s12913-022-07922-6 (PMC9047277; doi:10.1186/s12913-022-07922-6)
Supplement: Supplementary file 4 — Additional file 4: Supplementary table 4. Effect of catastrophic health expenditure on liquidity indicator. [file 12913_2022_7922_MOESM4_ESM.docx]

Supplementary table 4. Effect of catastrophic health expenditure on liquidity indicator

|  | | Odds Ratio | S.E. | P>\|z\| |
| --- | --- | --- | --- | --- |
| CHE | | 1.375 | 0.217 | 0.044 |
| Gender (Men) | | 0.843 | 0.177 | 0.419 |
| Age  (<39) | 40~64 | 1.081 | 0.290 | 0.770 |
|  | >65 | 0.672 | 0.124 | 0.033 |
| Educational level  (Elementary school) | Middle-high school | 1.600 | 0.374 | 0.044 |
|  | Greater than college | 1.336 | 0.350 | 0.268 |
| Marital (married) | Divorced, bereavement, separation | 1.115 | 0.428 | 0.777 |
|  | Unmarried | 1.593 | 0.409 | 0.069 |
| Employment  (Employee) | Employer/  Self-employed | 0.849 | 0.207 | 0.504 |
|  | Other | 0.883 | 0.443 | 0.805 |
|  | Unemployed | 1.774 | 0.388 | 0.009 |
| No. of household members (1) | 2 | 1.253 | 0.284 | 0.320 |
|  | 3 | 1.253 | 0.400 | 0.480 |
|  | >4 | 2.363 | 0.880 | 0.021 |
| Type of NHI  (Employee) | Employer/  Self-employed | 1.681 | 0.282 | 0.002 |
|  | Medical aid beneficiaries | 3.852 | 0.817 | 0.000 |
| Private insurance  (Insured) | Uninsured | 1.702 | 0.318 | 0.004 |
| Presence of disabled (No) | Yes | 0.721 | 0.160 | 0.141 |
| Presence of child (No) | Yes | 0.932 | 0.229 | 0.775 |
| Presence of elderly (No) | Yes | 0.725 | 0.188 | 0.216 |
| Constant | | 0.012 | 0.004 | 0.000 |
| N | | 4,802 | | |
| Log likelihood | | -897.2 | | |
| Pseudo R2 | | 0.067 | | |
