# Supplementary material for: Financial burden of catastrophic health expenditure on households with chronic diseases: financial ratio analysis
Source: BMC Health Serv Res. 2022 Apr 27;22:568. doi: 10.1186/s12913-022-07922-6 (PMC9047277; doi:10.1186/s12913-022-07922-6)
Supplement: Supplementary file 5 — Additional file 5: Supplementary table 5. Effect of catastrophic health expenditure on total living expenses. [file 12913_2022_7922_MOESM5_ESM.docx]

Supplementary table 5. Effect of catastrophic health expenditure on total living expenses

|  | | Coef. | S.E. | P>\|z\| |
| --- | --- | --- | --- | --- |
| CHE | | 0.058 | 0.014 | 0.000 |
| Gender (Men) | | -0.049 | 0.022 | 0.026 |
| Age  (<39) | 40~64 | 0.035 | 0.023 | 0.127 |
|  | >65 | -0.129 | 0.017 | 0.000 |
| Educational level  (Elementary school) | Middle-high school | -0.211 | 0.019 | 0.000 |
|  | Greater than college | -0.405 | 0.022 | 0.000 |
| Marital (married) | Divorced, bereavement, separation | -0.104 | 0.036 | 0.005 |
|  | Unmarried | -0.054 | 0.025 | 0.035 |
| Employment  (Employee) | Employer/  Self-employed | -0.024 | 0.019 | 0.220 |
|  | Other | -0.225 | 0.041 | 0.000 |
|  | Unemployed | -0.146 | 0.019 | 0.000 |
| No. of household members (1) | 2 | 0.402 | 0.023 | 0.000 |
|  | 3 | 0.742 | 0.030 | 0.000 |
|  | >4 | 1.042 | 0.035 | 0.000 |
| Type of NHI  (Employee) | Employer/  Self-employed | -0.050 | 0.015 | 0.001 |
|  | Medical aid beneficiaries | -0.234 | 0.024 | 0.000 |
| Private insurance  (Insured) | Uninsured | -0.260 | 0.017 | 0.000 |
| Presence of disabled (No) | Yes | -0.108 | 0.024 | 0.000 |
| Presence of child (No) | Yes | -0.079 | 0.023 | 0.001 |
| Presence of elderly (No) | Yes | -0.116 | 0.023 | 0.000 |
| Constant | | 7.869 | 0.035 | 0.000 |
| N | | 4,802 | | |
| F (20, 4781) | | 611.55 | | |
| Root MSE | | 0.431 | | |
| Adj R-squared | | 0.717 | | |
