# Supplementary material for: Financial burden of catastrophic health expenditure on households with chronic diseases: financial ratio analysis
Source: BMC Health Serv Res. 2022 Apr 27;22:568. doi: 10.1186/s12913-022-07922-6 (PMC9047277; doi:10.1186/s12913-022-07922-6)
Supplement: Supplementary file 6 — Additional file 6: Supplementary table 6. Effect of catastrophic health expenditure on disposable income. [file 12913_2022_7922_MOESM6_ESM.docx]

Supplementary table 6. Effect of catastrophic health expenditure on disposable income

|  | | Coef. | S.E. | P>\|z\| |
| --- | --- | --- | --- | --- |
| CHE | | -0.164 | 0.017 | 0.000 |
| Gender (Men) | | -0.070 | 0.025 | 0.006 |
| Age  (<39) | 40~64 | 0.009 | 0.027 | 0.717 |
|  | >65 | -0.113 | 0.020 | 0.000 |
| Educational level  (Elementary school) | Middle-high school | -0.180 | 0.022 | 0.000 |
|  | Greater than college | -0.339 | 0.025 | 0.000 |
| Marital (married) | Divorced, bereavement, separation | -0.071 | 0.042 | 0.093 |
|  | Unmarried | -0.032 | 0.029 | 0.272 |
| Employment  (Employee) | Employer/  Self-employed | -0.029 | 0.022 | 0.203 |
|  | Other | -0.255 | 0.047 | 0.000 |
|  | Unemployed | 0.226 | 0.022 | 0.000 |
| No. of household members (1) | 2 | 0.422 | 0.026 | 0.000 |
|  | 3 | 0.768 | 0.034 | 0.000 |
|  | >4 | 1.060 | 0.041 | 0.000 |
| Type of NHI  (Employee) | Employer/  Self-employed | -0.119 | 0.017 | 0.000 |
|  | Medical aid beneficiaries | -0.264 | 0.028 | 0.000 |
| Private insurance  (Insured) | Uninsured | -0.254 | 0.019 | 0.000 |
| Presence of disabled (No) | Yes | -0.079 | 0.027 | 0.004 |
| Presence of child (No) | Yes | -0.143 | 0.027 | 0.000 |
| Presence of elderly (No) | Yes | -0.082 | 0.026 | 0.002 |
| Constant | | 7.951 | 0.040 | 0.000 |
| N | | 4,783 | | |
| F (20, 4781) | | 462.07 | | |
| Root MSE | | 0.498 | | |
| Adj R-squared | | 0.658 | | |
