# Supplementary material for: Financial burden of catastrophic health expenditure on households with chronic diseases: financial ratio analysis
Source: BMC Health Serv Res. 2022 Apr 27;22:568. doi: 10.1186/s12913-022-07922-6 (PMC9047277; doi:10.1186/s12913-022-07922-6)
Supplement: Supplementary file 7 — Additional file 7: Supplementary table 7. Effect of catastrophic health expenditure on liquid assets. [file 12913_2022_7922_MOESM7_ESM.docx]

Supplementary table 7. Effect of catastrophic health expenditure on liquid assets

|  | | Coef. | S.E. | P>\|z\| |
| --- | --- | --- | --- | --- |
| CHE | | -0.383 | 0.069 | 0.000 |
| Gender (Men) | | -0.030 | 0.103 | 0.769 |
| Age  (<39) | 40~64 | -0.168 | 0.109 | 0.124 |
|  | >65 | -0.187 | 0.081 | 0.021 |
| Educational level  (Elementary school) | Middle-high school | -0.720 | 0.089 | 0.000 |
|  | Greater than college | -1.062 | 0.103 | 0.000 |
| Marital (married) | Divorced, bereavement, separation | -0.582 | 0.172 | 0.001 |
|  | Unmarried | -0.604 | 0.120 | 0.000 |
| Employment  (Employee) | Employer/  Self-employed | 0.320 | 0.092 | 0.001 |
|  | Other | -0.204 | 0.191 | 0.286 |
|  | Unemployed | -0.284 | 0.091 | 0.002 |
| No. of household members (1) | 2 | 0.220 | 0.109 | 0.043 |
|  | 3 | 0.344 | 0.140 | 0.014 |
|  | >4 | 0.423 | 0.168 | 0.012 |
| Type of NHI  (Employee) | Employer/  Self-employed | -0.399 | 0.071 | 0.000 |
|  | Medical aid beneficiaries | -2.004 | 0.114 | 0.000 |
| Private insurance  (Insured) | Uninsured | -0.479 | 0.080 | 0.000 |
| Presence of disabled (No) | Yes | -0.440 | 0.112 | 0.000 |
| Presence of child (No) | Yes | -0.614 | 0.109 | 0.000 |
| Presence of elderly (No) | Yes | -0.088 | 0.108 | 0.415 |
| Constant | | 8.438 | 0.164 | 0.000 |
| N | | 4,802 | | |
| F (20, 4781) | | 91.65 | | |
| Root MSE | | 2.021 | | |
| Adj R-squared | | 0.274 | | |
