# Supplementary material for: Financial burden of catastrophic health expenditure on households with chronic diseases: financial ratio analysis
Source: BMC Health Serv Res. 2022 Apr 27;22:568. doi: 10.1186/s12913-022-07922-6 (PMC9047277; doi:10.1186/s12913-022-07922-6)
Supplement: Supplementary file 8 — Additional file 8: Supplementary table 8. Effect of catastrophic health expenditure on total liability. [file 12913_2022_7922_MOESM8_ESM.docx]

Supplementary table 8. Effect of catastrophic health expenditure on total liability

|  | | Coef. | S.E. | P>\|z\| |
| --- | --- | --- | --- | --- |
| CHE | | 0.007 | 0.123 | 0.950 |
| Gender (Men) | | -0.195 | 0.186 | 0.293 |
| Age  (<39) | 40~64 | -0.069 | 0.196 | 0.722 |
|  | >65 | -0.681 | 0.146 | 0.000 |
| Educational level  (Elementary school) | Middle-high school | -0.205 | 0.160 | 0.199 |
|  | Greater than college | -1.110 | 0.185 | 0.000 |
| Marital (married) | Divorced, bereavement, separation | -0.439 | 0.309 | 0.156 |
|  | Unmarried | 0.241 | 0.216 | 0.265 |
| Employment  (Employee) | Employer/  Self-employed | -0.555 | 0.165 | 0.001 |
|  | Other | -0.415 | 0.343 | 0.227 |
|  | Unemployed | -0.422 | 0.164 | 0.010 |
| No. of household members (1) | 2 | 0.586 | 0.195 | 0.003 |
|  | 3 | 1.697 | 0.251 | 0.000 |
|  | >4 | 2.250 | 0.301 | 0.000 |
| Type of NHI  (Employee) | Employer/  Self-employed | 0.535 | 0.127 | 0.000 |
|  | Medical aid beneficiaries | 0.254 | 0.204 | 0.214 |
| Private insurance  (Insured) | Uninsured | -0.396 | 0.144 | 0.006 |
| Presence of disabled (No) | Yes | -0.576 | 0.201 | 0.004 |
| Presence of child (No) | Yes | 0.030 | 0.196 | 0.877 |
| Presence of elderly (No) | Yes | -0.521 | 0.193 | 0.007 |
| Constant | | 3.767 | 0.294 | 0.000 |
| N | | 4,802 | | |
| F (20, 4781) | | 54.75 | | |
| Root MSE | | 3.621 | | |
| Adj R-squared | | 0.182 | | |
