# Supplementary material for: Financial burden of catastrophic health expenditure on households with chronic diseases: financial ratio analysis
Source: BMC Health Serv Res. 2022 Apr 27;22:568. doi: 10.1186/s12913-022-07922-6 (PMC9047277; doi:10.1186/s12913-022-07922-6)
Supplement: Supplementary file 9 — Additional file 9: Supplementary table 9. Effect of catastrophic health expenditure on total assets. [file 12913_2022_7922_MOESM9_ESM.docx]

Supplementary table 9. Effect of catastrophic health expenditure on total assets

|  | | Coef. | S.E. | P>\|z\| |
| --- | --- | --- | --- | --- |
| CHE | | -0.146 | 0.056 | 0.010 |
| Gender (Men) | | -0.104 | 0.085 | 0.220 |
| Age  (<39) | 40~64 | -0.120 | 0.090 | 0.182 |
|  | >65 | -0.296 | 0.067 | 0.000 |
| Educational level  (Elementary school) | Middle-high school | -0.450 | 0.073 | 0.000 |
|  | Greater than college | -0.874 | 0.085 | 0.000 |
| Marital (married) | Divorced, bereavement, separation | -0.946 | 0.142 | 0.000 |
|  | Unmarried | -0.769 | 0.099 | 0.000 |
| Employment  (Employee) | Employer/  Self-employed | 0.705 | 0.075 | 0.000 |
|  | Other | -0.067 | 0.158 | 0.669 |
|  | Unemployed | -0.118 | 0.075 | 0.117 |
| No. of household members (1) | 2 | 0.308 | 0.089 | 0.001 |
|  | 3 | 0.532 | 0.115 | 0.000 |
|  | >4 | 0.738 | 0.138 | 0.000 |
| Type of NHI  (Employee) | Employer/  Self-employed | -0.322 | 0.058 | 0.000 |
|  | Medical aid beneficiaries | -1.701 | 0.094 | 0.000 |
| Private insurance  (Insured) | Uninsured | -0.299 | 0.066 | 0.000 |
| Presence of disabled (No) | Yes | -0.777 | 0.092 | 0.000 |
| Presence of child (No) | Yes | -0.571 | 0.090 | 0.000 |
| Presence of elderly (No) | Yes | 0.175 | 0.089 | 0.049 |
| Constant | | 10.044 | 0.135 | 0.000 |
| N | | 4,802 | | |
| F (20, 4781) | | 143.89 | | |
| Root MSE | | 1.666 | | |
| Adj R-squared | | 0.373 | | |
