# Supplementary material for: Financial burden of catastrophic health expenditure on households with chronic diseases: financial ratio analysis
Source: BMC Health Serv Res. 2022 Apr 27;22:568. doi: 10.1186/s12913-022-07922-6 (PMC9047277; doi:10.1186/s12913-022-07922-6)
Supplement: Supplementary file 10 — Additional file 10: Supplementary table 10. Effect of catastrophic health expenditure on non-liquid assets. [file 12913_2022_7922_MOESM10_ESM.docx]

Supplementary table 10. Effect of catastrophic health expenditure on non-liquid assets

|  | | Coef. | S.E. | P>\|z\| |
| --- | --- | --- | --- | --- |
| CHE | | -0.036 | 0.049 | 0.459 |
| Gender (Men) | | 0.120 | 0.074 | 0.107 |
| Age  (<39) | 40~64 | 0.031 | 0.076 | 0.684 |
|  | >65 | -0.037 | 0.058 | 0.516 |
| Educational level  (Elementary school) | Middle-high school | -0.496 | 0.062 | 0.000 |
|  | Greater than college | -0.863 | 0.072 | 0.000 |
| Marital (married) | Divorced, bereavement, separation | -1.052 | 0.123 | 0.000 |
|  | Unmarried | -0.716 | 0.085 | 0.000 |
| Employment  (Employee) | Employer/  Self-employed | 0.666 | 0.063 | 0.000 |
|  | Other | 0.038 | 0.139 | 0.781 |
|  | Unemployed | 0.124 | 0.064 | 0.053 |
| No. of household members (1) | 2 | 0.077 | 0.078 | 0.326 |
|  | 3 | 0.316 | 0.098 | 0.001 |
|  | >4 | 0.506 | 0.117 | 0.000 |
| Type of NHI  (Employee) | Employer/  Self-employed | -0.287 | 0.050 | 0.000 |
|  | Medical aid beneficiaries | -1.973 | 0.084 | 0.000 |
| Private insurance  (Insured) | Uninsured | -0.138 | 0.056 | 0.014 |
| Presence of disabled (No) | Yes | -0.901 | 0.081 | 0.000 |
| Presence of child (No) | Yes | -0.372 | 0.075 | 0.000 |
| Presence of elderly (No) | Yes | 0.349 | 0.074 | 0.000 |
| Constant | | 9.736 | 0.115 | 0.000 |
| N | | 4,295 | | |
| F (20, 4781) | | 137.24 | | |
| Root MSE | | 1.357 | | |
| Adj R-squared | | 0.388 | | |
