# Supplementary material for: Financial burden of catastrophic health expenditure on households with chronic diseases: financial ratio analysis
Source: BMC Health Serv Res. 2022 Apr 27;22:568. doi: 10.1186/s12913-022-07922-6 (PMC9047277; doi:10.1186/s12913-022-07922-6)
Supplement: Supplementary file 11 — Additional file 11: Supplementary table 11. Effect of catastrophic health expenditure on total living expenses without OOP [file 12913_2022_7922_MOESM11_ESM.docx]

Supplementary table 11. Effect of catastrophic health expenditure on total living expenses without OOP

|  | | Coef. | S.E. | P>\|z\| |
| --- | --- | --- | --- | --- |
| CHE | | -0.096 | 0.014 | 0.000 |
| Gender (Men) | | -0.131 | 0.022 | 0.000 |
| Age  (<39) | 40~64 | -0.010 | 0.023 | 0.652 |
|  | >65 | -0.151 | 0.017 | 0.000 |
| Educational level  (Elementary school) | Middle-high school | -0.216 | 0.019 | 0.000 |
|  | Greater than college | -0.412 | 0.022 | 0.000 |
| Marital (married) | Divorced, bereavement, separation | -0.060 | 0.036 | 0.102 |
|  | Unmarried | 0.088 | 0.025 | 0.001 |
| Employment  (Employee) | Employer/  Self-employed | 0.000 | 0.019 | 0.981 |
|  | Other | -0.242 | 0.040 | 0.000 |
|  | Unemployed | -0.167 | 0.019 | 0.000 |
| No. of household members (1) | 2 | 0.452 | 0.232 | 0.000 |
|  | 3 | 0.818 | 0.298 | 0.000 |
|  | >4 | 1.136 | 0.035 | 0.000 |
| Type of NHI  (Employee) | Employer/  Self-employed | -0.057 | 0.015 | 0.000 |
|  | Medical aid beneficiaries | -0.216 | 0.024 | 0.000 |
| Private insurance  (Insured) | Uninsured | -0.301 | 0.017 | 0.000 |
| Presence of disabled (No) | Yes | -0.120 | 0.023 | 0.000 |
| Presence of child (No) | Yes | -0.152 | 0.023 | 0.000 |
| Presence of elderly (No) | Yes | -0.170 | 0.022 | 0.000 |
| Constant | | 12.783 | 0.034 | 0.000 |
| N | | 4,802 | | |
| F (20, 4781) | | 697.10 | | |
| Root MSE | | 0.43 | | |
| Adj R-squared | | 0.743 | | |
