# Supplementary material for: Financial burden of catastrophic health expenditure on households with chronic diseases: financial ratio analysis
Source: BMC Health Serv Res. 2022 Apr 27;22:568. doi: 10.1186/s12913-022-07922-6 (PMC9047277; doi:10.1186/s12913-022-07922-6)
Supplement: Supplementary file 12 — Additional file 12: Supplementary table 12. Effect of catastrophic health expenditure on earned income. [file 12913_2022_7922_MOESM12_ESM.docx]

Supplementary table 12. Effect of catastrophic health expenditure on earned income

|  | | Coef. | S.E. | P>\|z\| |
| --- | --- | --- | --- | --- |
| CHE | | -0.385 | 0.044 | 0.000 |
| Gender (Men) | | -0.199 | 0.063 | 0.002 |
| Age  (<39) | 40~64 | 0.052 | 0.051 | 0.307 |
|  | >65 | -0.231 | 0.056 | 0.000 |
| Educational level  (Elementary school) | Middle-high school | -0.141 | 0.045 | 0.002 |
|  | Greater than college | -0.265 | 0.058 | 0.000 |
| Marital (married) | Divorced, bereavement, separation | 0.325 | 0.086 | 0.000 |
|  | Unmarried | 0.214 | 0.066 | 0.001 |
| Employment  (Employee) | Employer/  Self-employed | -0.875 | 0.048 | 0.000 |
|  | Other | -0.843 | 0.088 | 0.000 |
|  | Unemployed | -0.561 | 0.046 | 0.000 |
| No. of household members (1) | 2 | 0.639 | 0.063 | 0.000 |
|  | 3 | 1.253 | 0.073 | 0.000 |
|  | >4 | 1.631 | 0.083 | 0.000 |
| Type of NHI  (Employee) | Employer/  Self-employed | -0.262 | 0.040 | 0.000 |
|  | Medical aid beneficiaries | -0.753 | 0.089 | 0.000 |
| Private insurance  (Insured) | Uninsured | -0.330 | 0.045 | 0.000 |
| Presence of disabled (No) | Yes | -0.174 | 0.075 | 0.021 |
| Presence of child (No) | Yes | -0.310 | 0.049 | 0.000 |
| Presence of elderly (No) | Yes | -0.350 | 0.049 | 0.000 |
| Constant | | 7.450 | 0.081 | 0.000 |
| N | | 2,467 | | |
| F (20, 4781) | | 191.45 | | |
| Root MSE | | 0.820 | | |
| Adj R-squared | | 0.607 | | |
