# Supplementary material for: Financial burden of catastrophic health expenditure on households with chronic diseases: financial ratio analysis
Source: BMC Health Serv Res. 2022 Apr 27;22:568. doi: 10.1186/s12913-022-07922-6 (PMC9047277; doi:10.1186/s12913-022-07922-6)
Supplement: Supplementary file 13 — Additional file 13: Supplementary table 13. Effect of catastrophic health expenditure on business income. [file 12913_2022_7922_MOESM13_ESM.docx]

Supplementary table 13. Effect of catastrophic health expenditure on business income

|  | | Coef. | S.E. | P>\|z\| |
| --- | --- | --- | --- | --- |
| CHE | | -0.418 | 0.075 | 0.000 |
| Gender (Men) | | -0.334 | 0.142 | 0.019 |
| Age  (<39) | 40~64 | 0.070 | 0.126 | 0.578 |
|  | >65 | -0.339 | 0.087 | 0.000 |
| Educational level  (Elementary school) | Middle-high school | -0.244 | 0.109 | 0.025 |
|  | Greater than college | -0.509 | 0.122 | 0.000 |
| Marital (married) | Divorced, bereavement, separation | 0.242 | 0.248 | 0.330 |
|  | Unmarried | -0.283supplementary | 0.150 | 0.060 |
| Employment  (Employee) | Employer/  Self-employed | 1.524 | 0.107 | 0.000 |
|  | Other | 0.441 | 0.224 | 0.049 |
|  | Unemployed | -0.028 | 0.131 | 0.831 |
| No. of household members (1) | 2 | 0.233 | 0.147 | 0.113 |
|  | 3 | 0.550 | 0.173 | 0.002 |
|  | >4 | 0.710 | 0.201 | 0.000 |
| Type of NHI  (Employee) | Employer/  Self-employed | 0.199 | 0.072 | 0.006 |
|  | Medical aid beneficiaries | -0.645 | 0.236 | 0.006 |
| Private insurance  (Insured) | Uninsured | -0.542 | 0.086 | 0.000 |
| Presence of disabled (No) | Yes | -0.532 | 0.147 | 0.000 |
| Presence of child (No) | Yes | 0.080 | 0.120 | 0.504 |
| Presence of elderly (No) | Yes | -0.517 | 0.118 | 0.000 |
| Constant | | 6.057 | 0.216 | 0.000 |
| N | | 1,627 | | |
| F (20, 4781) | | 101.89 | | |
| Root MSE | | 1.2907 | | |
| Adj R-squared | | 0.553 | | |
