# Supplementary material for: Financial burden of catastrophic health expenditure on households with chronic diseases: financial ratio analysis
Source: BMC Health Serv Res. 2022 Apr 27;22:568. doi: 10.1186/s12913-022-07922-6 (PMC9047277; doi:10.1186/s12913-022-07922-6)
Supplement: Supplementary file 14 — Additional file 14: Supplementary table 14. Effect of catastrophic health expenditure on property income. [file 12913_2022_7922_MOESM14_ESM.docx]

Supplementary table 14. Effect of catastrophic health expenditure on property income

|  | | Coef. | S.E. | P>\|z\| |
| --- | --- | --- | --- | --- |
| CHE | | -0.106 | 0.087 | 0.228 |
| Gender (Men) | | -0.106 | 0.166 | 0.524 |
| Age  (<39) | 40~64 | 0.073 | 0.159 | 0.645 |
|  | >65 | 0.035 | 0.102 | 0.728 |
| Educational level  (Elementary school) | Middle-high school | -0.357 | 0.109 | 0.001 |
|  | Greater than college | -0.963 | 0.127 | 0.000 |
| Marital (married) | Divorced, bereavement, separation | -0.874 | 0.271 | 0.001 |
|  | Unmarried | -0.001 | 0.188 | 0.993 |
| Employment  (Employee) | Employer/  Self-employed | -0.662 | 0.116 | 0.000 |
|  | Other | -0.617 | 0.271 | 0.023 |
|  | Unemployed | 0.284 | 0.122 | 0.021 |
| No. of household members (1) | 2 | 0.395 | 0.176 | 0.025 |
|  | 3 | 0.399 | 0.208 | 0.055 |
|  | >4 | 0.604 | 0.235 | 0.010 |
| Type of NHI  (Employee) | Employer/  Self-employed | 0.342 | 0.091 | 0.000 |
|  | Medical aid beneficiaries | -1.108 | 0.370 | 0.003 |
| Private insurance  (Insured) | Uninsured | 0.006 | 0.101 | 0.949 |
| Presence of disabled (No) | Yes | -0.871 | 0.218 | 0.000 |
| Presence of child (No) | Yes | -0.881 | 0.154 | 0.000 |
| Presence of elderly (No) | Yes | -0.031 | 0.154 | 0.838 |
| Constant | | 5.172 | 0.244 | 0.000 |
| N | | 1,797 | | |
| F (20, 4781) | | 16.75 | | |
| Root MSE | | 1.600 | | |
| Adj R-squared | | 0.149 | | |
