# Supplementary material for: Financial burden of catastrophic health expenditure on households with chronic diseases: financial ratio analysis
Source: BMC Health Serv Res. 2022 Apr 27;22:568. doi: 10.1186/s12913-022-07922-6 (PMC9047277; doi:10.1186/s12913-022-07922-6)
Supplement: Supplementary file 15 — Additional file 15: Supplementary table 15.Effect of catastrophic health expenditure on private transfer income. [file 12913_2022_7922_MOESM15_ESM.docx]

Supplementary table 15. Effect of catastrophic health expenditure on private transfer income

|  | | Coef. | S.E. | P>\|z\| |
| --- | --- | --- | --- | --- |
| CHE | | 0.410 | 0.048 | 0.000 |
| Gender (Men) | | 0.293 | 0.073 | 0.000 |
| Age  (<39) | 40~64 | 0.028 | 0.077 | 0.717 |
|  | >65 | 0.248 | 0.057 | 0.000 |
| Educational level  (Elementary school) | Middle-high school | -0.196 | 0.063 | 0.002 |
|  | Greater than college | -0.255 | 0.073 | 0.001 |
| Marital (married) | Divorced, bereavement, separation | -0.830 | 0.122 | 0.000 |
|  | Unmarried | -0.254 | 0.085 | 0.003 |
| Employment  (Employee) | Employer/  Self-employed | 0.289 | 0.065 | 0.000 |
|  | Other | 0.212 | 0.135 | 0.117 |
|  | Unemployed | 0.460 | 0.064 | 0.000 |
| No. of household members (1) | 2 | -0.007 | 0.077 | 0.926 |
|  | 3 | -0.489 | 0.099 | 0.000 |
|  | >4 | -0.809 | 0.119 | 0.000 |
| Type of NHI  (Employee) | Employer/  Self-employed | -0.285 | 0.050 | 0.000 |
|  | Medical aid beneficiaries | -1.191 | 0.081 | 0.000 |
| Private insurance  (Insured) | Uninsured | -0.029 | 0.057 | 0.610 |
| Presence of disabled (No) | Yes | -0.097 | 0.079 | 0.224 |
| Presence of child (No) | Yes | 0.433 | 0.077 | 0.000 |
| Presence of elderly (No) | Yes | 0.585 | 0.076 | 0.000 |
| Constant | | 4.791 | 0.116 | 0.000 |
| N | | 4,781 | | |
| F (20, 4781) | | 67.27 | | |
| Root MSE | | 1.428 | | |
| Adj R-squared | | 0.217 | | |
