# Supplementary material for: Financial burden of catastrophic health expenditure on households with chronic diseases: financial ratio analysis
Source: BMC Health Serv Res. 2022 Apr 27;22:568. doi: 10.1186/s12913-022-07922-6 (PMC9047277; doi:10.1186/s12913-022-07922-6)
Supplement: Supplementary file 16 — Additional file 16: Supplementary table 16. Effect of catastrophic health expenditure on public transfer income. [file 12913_2022_7922_MOESM16_ESM.docx]

Supplementary table 16. Effect of catastrophic health expenditure on public transfer income

|  | | Coef. | S.E. | P>\|z\| |
| --- | --- | --- | --- | --- |
| CHE | | -0.143 | 0.030 | 0.000 |
| Gender (Men) | | -0.077 | 0.046 | 0.094 |
| Age  (<39) | 40~64 | -0.240 | 0.049 | 0.000 |
|  | >65 | -0.199 | 0.035 | 0.000 |
| Educational level  (Elementary school) | Middle-high school | -0.252 | 0.043 | 0.000 |
|  | Greater than college | -0.344 | 0.048 | 0.000 |
| Marital (married) | Divorced, bereavement, separation | -0.302 | 0.081 | 0.000 |
|  | Unmarried | -0.099 | 0.054 | 0.070 |
| Employment  (Employee) | Employer/  Self-employed | 0.140 | 0.043 | 0.001 |
|  | Other | 0.182 | 0.083 | 0.028 |
|  | Unemployed | 0.269 | 0.042 | 0.000 |
| No. of household members (1) | 2 | 0.350 | 0.050 | 0.000 |
|  | 3 | 0.399 | 0.065 | 0.000 |
|  | >4 | 0.217 | 0.081 | 0.007 |
| Type of NHI  (Employee) | Employer/  Self-employed | -0.104 | 0.032 | 0.001 |
|  | Medical aid beneficiaries | 0.305 | 0.049 | 0.000 |
| Private insurance  (Insured) | Uninsured | -0.046 | 0.035 | 0.196 |
| Presence of disabled (No) | Yes | 0.351 | 0.047 | 0.000 |
| Presence of child (No) | Yes | -0.063 | 0.054 | 0.243 |
| Presence of elderly (No) | Yes | 0.562 | 0.050 | 0.000 |
| Constant | | 5.749 | 0.078 | 0.000 |
| N | | 4,112 | | |
| F (20, 4781) | | 45.93 | | |
| Root MSE | | 0.850 | | |
| Adj R-squared | | 0.179 | | |
