# Supplementary material for: Financial burden of catastrophic health expenditure on households with chronic diseases: financial ratio analysis
Source: BMC Health Serv Res. 2022 Apr 27;22:568. doi: 10.1186/s12913-022-07922-6 (PMC9047277; doi:10.1186/s12913-022-07922-6)
Supplement: Supplementary file 17 — Additional file 17: Supplementary table 17. Effect of catastrophic health expenditure on other income. [file 12913_2022_7922_MOESM17_ESM.docx]

Supplementary table 17. Effect of catastrophic health expenditure on other income

|  | | Coef. | S.E. | P>\|z\| |
| --- | --- | --- | --- | --- |
| CHE | | 0.309 | 0.064 | 0.000 |
| Gender (Men) | | -0.073 | 0.097 | 0.451 |
| Age  (<39) | 40~64 | 0.042 | 0.101 | 0.675 |
|  | >65 | -0.149 | 0.076 | 0.049 |
| Educational level  (Elementary school) | Middle-high school | -0.106 | 0.083 | 0.202 |
|  | Greater than college | -0.209 | 0.096 | 0.031 |
| Marital (married) | Divorced, bereavement, separation | -0.072 | 0.160 | 0.650 |
|  | Unmarried | -0.125 | 0.112 | 0.264 |
| Employment  (Employee) | Employer/  Self-employed | -0.273 | 0.085 | 0.001 |
|  | Other | -0.176 | 0.177 | 0.320 |
|  | Unemployed | -0.344 | 0.085 | 0.000 |
| No. of household members (1) | 2 | 0.382 | 0.101 | 0.000 |
|  | 3 | 0.922 | 0.130 | 0.000 |
|  | >4 | 0.959 | 0.156 | 0.000 |
| Type of NHI  (Employee) | Employer/  Self-employed | -0.135 | 0.066 | 0.042 |
|  | Medical aid beneficiaries | -0.214 | 0.106 | 0.045 |
| Private insurance  (Insured) | Uninsured | -0.504 | 0.075 | 0.000 |
| Presence of disabled (No) | Yes | -0.148 | 0.104 | 0.156 |
| Presence of child (No) | Yes | -0.588 | 0.101 | 0.000 |
| Presence of elderly (No) | Yes | -0.157 | 0.100 | 0.117 |
| Constant | | 2.685 | 0.152 | 0.000 |
| N | | 4,709 | | |
| F (20, 4781) | | 36.76 | | |
| Root MSE | | 1.865 | | |
| Adj R-squared | | 0.131 | | |
