# Supplementary material for: Financial burden of catastrophic health expenditure on households with chronic diseases: financial ratio analysis
Source: BMC Health Serv Res. 2022 Apr 27;22:568. doi: 10.1186/s12913-022-07922-6 (PMC9047277; doi:10.1186/s12913-022-07922-6)
Supplement: Supplementary file 18 — Additional file 18. irb report. [file 12913_2022_7922_MOESM18_ESM.pdf]

생명윤리위원회(IRB) 심사결과 통지서

다음과 같이 심사결과를 통보합니다.

문서번호	제2021-002호		발송일자	2021.03.05.		
연구과제명	한국복지패널 구축 및 운영					
과제번호	[일반 21-016-00]					
연구책임자	(성명) 정은희 (소속) 한국보건사회연구원 (직위) 부연구위원					
심사일자	2021.02.17.~2021.03.05.		심사형태	정규심사		
심사결과	승인 ☐	시정승인 ■		재심의 ☐	부결 ☐	
총 연구기간	2021. 01. 01. ~ 2021. 12. 31.					
IRB 연구승인 유효기간	2021.03.05부터 2022.03.04까지	· 총 신청 연구기간이 IRB 연구승인 유효기간을 초과할 경우, 유효기간 만료 이전에 '지속심사' 승인을 받아야 연구지속 진행이 가능합니다.<br>· 연구종료 시 종료보고를 하여주시기 바랍니다.				
심사내용						
심사의견	☐ 정규(대면)심사 결과, 검토의견에 대한 보완 및 수정이 적절히 이루어졌다고 판단하여 심의결과를 '시정승인'으로 결정함.					

모든 연구자들은 아래의 사항을 준수하여야 합니다.

1. 연구자께서는 제출하신 계획서에 따라 연구를 수행하여야 하며, 이와 다르게 연구를 진행하실 경우 다시 심의를 진행하셔야 함을 유의하시기 바랍니다.
2. 위원회의 요구가 있을 때에는 연구의 진행과 관련된 보고를 위원회에 제출하여야 합니다.
3. 연구윤리를 위하여 관련부처가 필요시 조사 및 감독 차원에서 현장점검을 실시할 수 있습니다.

※ 연구책임자는 본 위원회의 심사결과에 대하여 이의가 있을 경우, 심사결과 통지일로부터 2주 이내에 서면으로 이의신청을 할 수 있습니다. 단, 동일 사안에 대하여 2회 이상의 재심은 하지 않습니다.

2021년 03월 08일

한국보건사회연구원 생명윤리위원회 (직인)
