# Supplementary material for: Financial burden of catastrophic health expenditure on households with chronic diseases: financial ratio analysis
Source: BMC Health Serv Res. 2022 Apr 27;22:568. doi: 10.1186/s12913-022-07922-6 (PMC9047277; doi:10.1186/s12913-022-07922-6)
Supplement: Supplementary file 19 — Additional file 19. Thedatabase link of Korea Welfare Panel Study (KoWePS). [file 12913_2022_7922_MOESM19_ESM.docx]

The database link of Korea Welfare Panel Study (KoWePS)

<https://www.koweps.re.kr:442/main.do>
